# Hip position may be associated with susceptibility to Rommens type IIIa fragility fractures of the pelvis during lateral falls: a quantitative computed tomography-based finite element analysis

**DOI:** 10.1186/s12891-026-10164-w

**Published:** 2026-07-04

**Authors:** Kunihiko Arakawa, Takashi Suzuki, Yoshinobu Watanabe, Takahiro Inui, Gen Sasaki, Akifumi Honda, Hirotaka Kawano

**Affiliations:** 1 Department of Orthopaedic Surgery, Teikyo University School of Medicine, 2-11-1, Kaga, Itabashi, Tokyo 173-8605 Japan; 2 Department of Emergency Medicine, Teikyo University School of Medicine, 2-11-1, Kaga, Itabashi, Tokyo 173-8605 Japan

**Keywords:** Fragility fracture, Pelvic ring fracture, Rommens classification, Injury mechanism, Quantitative computed tomography-based finite element analysis, Osteoporosis

## Abstract

**Background:**

The incidence of fragility fractures of the pelvis (FFP) has increased, significantly impacting healthcare systems. While the Rommens classification comprehensively categorizes FFP, the injury mechanism—particularly in type IIIa FFP, which involves an externally rotated iliac wing fracture and a pubic rami fracture—remains unclear. This study investigated the injury mechanism of type IIIa FFP using a quantitative computed tomography-based finite element analysis (QCT/FEA).

**Methods:**

A patient-specific QCT/FEA model of the hemi-pelvis and femur on the healthy side was developed using CT data from a patient with type IIIa FFP. The sacrum was fully constrained, whereas the pubic symphysis and the femur were partially constrained. Direct loading conditions were applied from the iliac wing, the greater trochanter, and the ischial tuberosity. Indirect loading conditions were applied from the greater trochanter under various hip positions (0º to 90º flexion at 15º intervals). Compressive and tensile failure clusters were evaluated under nonlinear analyses. Loading was increased in 40 N increments up to 2000 N. Additionally, force–displacement curves and external constraint forces were measured in models with 3-mm forced displacement.

**Results:**

Direct horizontal loading from the iliac wing produced clusters of tensile failure elements in the posterior ilium. In contrast, indirect loading transmitted through the hip joint along the axis of the femoral neck predominantly produced clusters of compressive failure elements in the posterior ilium, particularly in the 30º–60º flexion models. The lowest peak load in the force–displacement curve was observed in the 60º flexion with internal rotation model. These findings suggest that different load transmission pathways produce distinct failure distributions during lateral compression loading.

**Conclusion:**

The injury mechanism of Rommens type IIIa FFP may involve both direct lateral impact to the ilium and indirect load transmission through the hip joint. In addition, hip position may influence load transmission through the hip joint during lateral falls.

## Introduction

Recently, the incidence of fragility fractures of the pelvis (FFP) among elderly individuals has increased rapidly due to bone fragility caused by osteoporosis. With the aging population, the number of patients with FFP can triple from 2005 to 2030 [1, 2]. This increase can have a significant impact on the healthcare system and worsen the life expectancy of elderly individuals [3, 4].

The Rommens and Hofmann classification systematically categorized FFP according to the degree of instability and facilitated the determination of appropriate treatment strategies [5, 6]. Recent observational studies have shown that Rommens type III FFP, particularly type IIIa, is associated with high mortality and reduced mobility rates [7, 8]. Rommens type IIIa FFP is characterized by an externally rotated iliac wing fracture combined with pubic rami fractures, which is more likely to be an indication for surgery [2, 7]. The morphology of the fracture does not correspond to the Young and Burgess classification of high-energy pelvic ring fractures, which classifies fractures according to injury mechanisms and helps guide management strategies. Hence, the mechanism associated with the development of this type of fracture remains unknown [9–12]. Moreover, the mechanism of injury in Rommens type IIIa fracture should be elucidated to prevent this type of fracture, improve intraoperative reduction techniques, and identify risk factors.

Several biomechanical analyses using quantitative computed tomography-based finite element analysis (QCT/FEA) models have identified the mechanism of injury in FFP [13–17]. Nevertheless, data regarding these mechanisms remain limited. Moreover, there are no reports investigating the mechanism related to the development of Rommens type IIIa FFP nor reflecting the bone morphology of patients with FFP. Therefore, the current study aimed to investigate the biomechanical mechanisms associated with Rommens type IIIa FFP using a patient-specific QCT/FEA model, with particular focus on hip-position-dependent loading conditions during lateral falls. The findings of this study could contribute to improving preventive strategies, refining surgical approaches, and enhancing postoperative care for elderly patients with pelvic fragility fractures.

## Materials and methods

### Ethics approval and consent to participate

This study was approved by Teikyo University Medical Research Ethics Committee of Teikyo University School of Medicine, Tokyo, Japan (approval number: 22–093). As a preliminary investigation for a larger-scale case study, individual patient consent was obtained using the opt-out method, which was also approved by the ethics committee.

### Subject

**Fig. 1:**
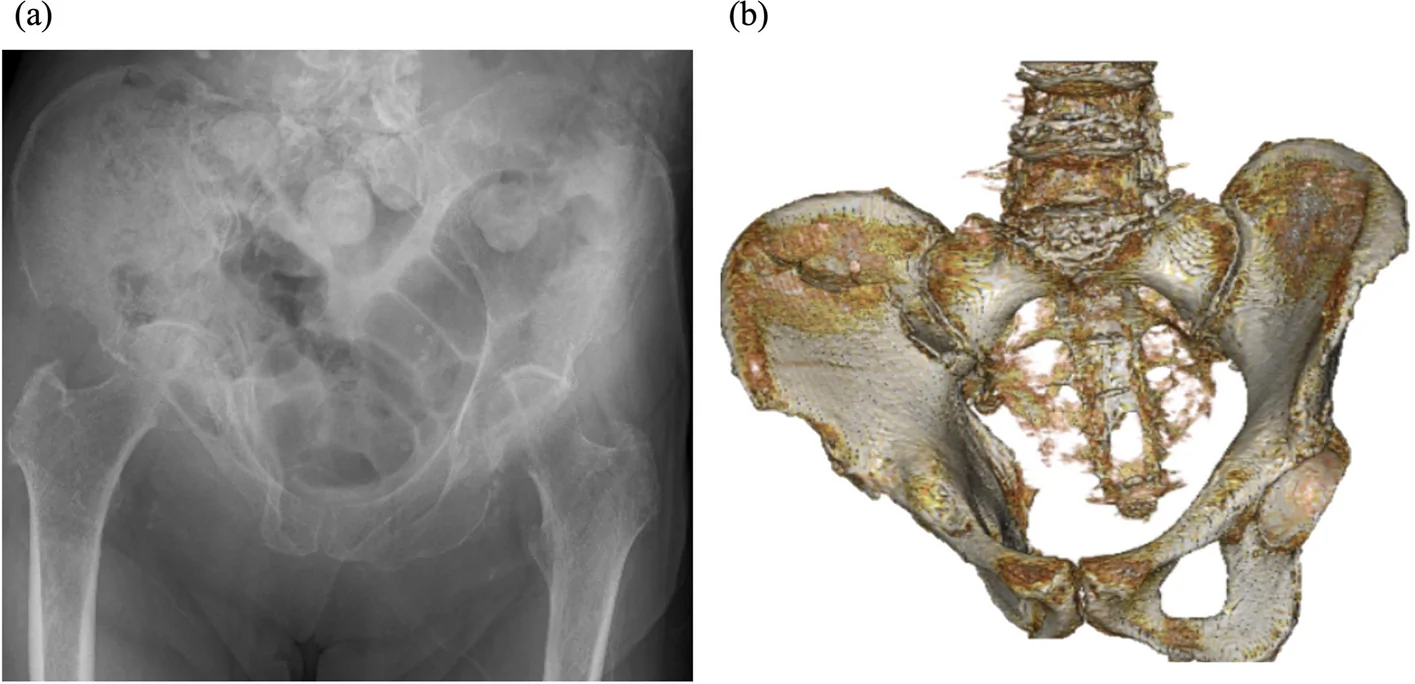
Original radiographic images. The (**a**) antero-posterior radiograph and (**b**) three-dimensional computed tomography scan images of a patient with Rommens type IIIa FFP were used to create QCT/FEA models

The current study included a female patient in her 80 s with Rommens type IIIa FFP. The patient sustained an injury after falling posterior-laterally from the standing position. The injury patterns were reported by the patient herself and corroborated by family members who directly witnessed the incident. Radiography and computed tomography (CT) scan were performed immediately after the patient’s visit to our trauma center (Fig. 1). Images were acquired using a CT scan system (Aquilion ONE, Canon Medical Systems Inc., Tochigi, Japan), with slices of 0.5-mm thickness.

### Quantitative CT-based finite element analysis

**Fig. 2:**
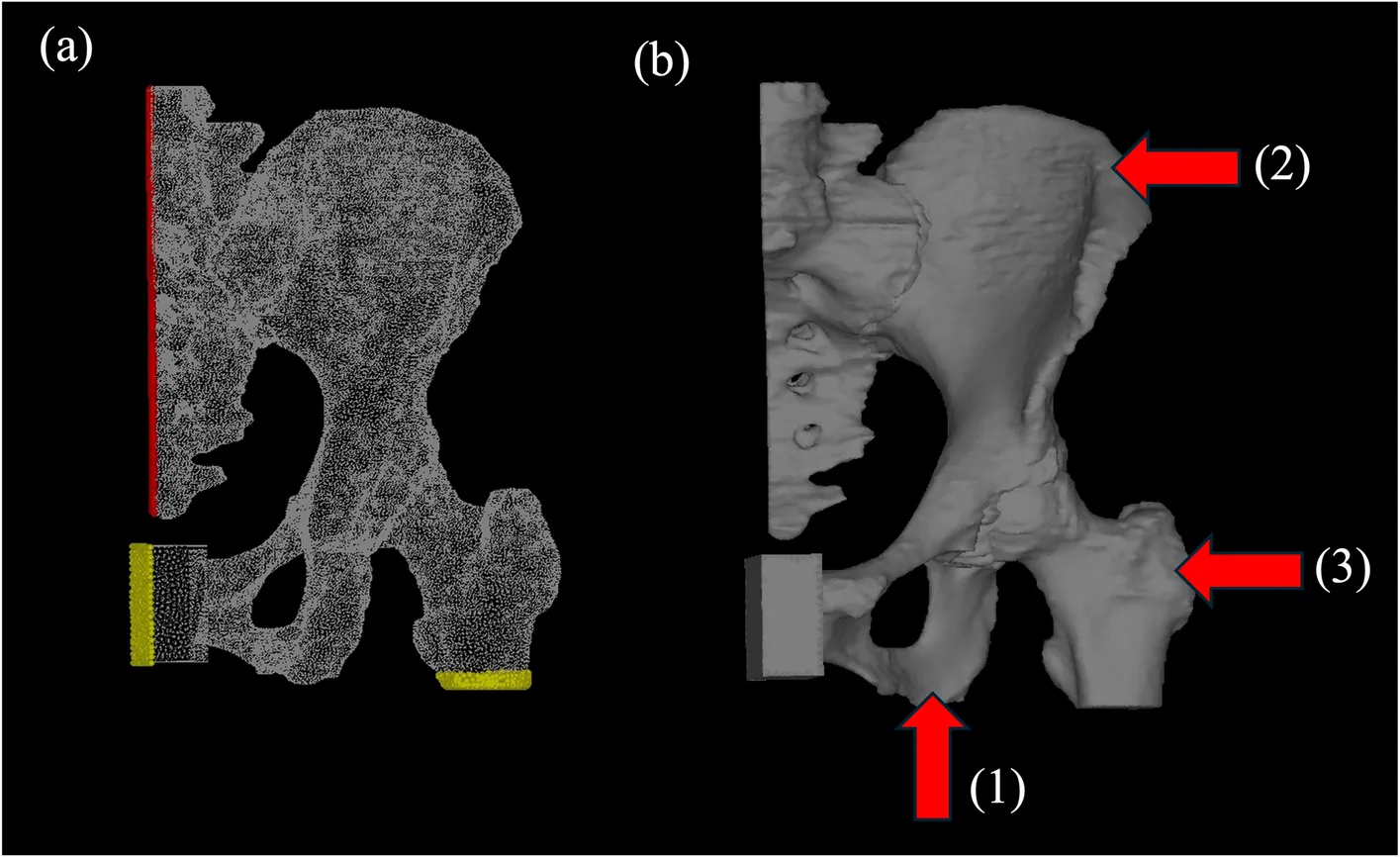
Overview of the QCT/FEA model, constraint conditions, and loading conditions in analysis 1. **a** The sacrum was completely constrained at the center (red). The femur and the pubic symphysis were partially constrained (yellow). **b** Loading conditions of direct force in analysis 1. (1) Vertical load from the ischial tuberosity. (2) Horizontal load from the iliac wing. (3) Horizontal load from the greater trochanter

The QCT/FEA software (MECHANICAL FINDER version 12.0 Extended Edition, Research Center of Computational Mechanics, Inc., Tokyo, Japan) was utilized for segmentation, meshing, assignment of material properties, setting of boundary condition, QCT/FEA, and post-processing. The CT DICOM data were imported into the MECHANICAL FINDER software to establish a three-dimensional QCT/FEA model. The non-fracture side of the hemi-pelvis and proximal femur model was created (Fig. 2a). The calibrated hydroxyapatite equivalent density was obtained with the following equation using Hounsfield units (HU) without a phantom:

$$\begin{aligned} \uprho\mathrm{HA}\;\lbrack\mathrm{mg}/\mathrm{cm}3\rbrack\;&=\;0.945\;\times\;\lbrack\mathrm{CT}\;\mathrm{value}\;(\mathrm{HU})\rbrack\\ &\quad+\;1.35 \end{aligned}$$

The mesh was made using first-order tetrahedral elements with a general size of 2.0 mm and a minimum size of 0.2 mm. The 0.3-mm shell elements were placed on the surface of the bone model accounting for the partial volume effect in the material nonlinear analysis [18, 19]. Mesh convergence was investigated using the 2.0 − 4.0-mm general side models, thereby maintaining the general size-to-minimum size ratio constant. Using the same material properties and boundary conditions, each model was analyzed. The total strain energy was evaluated. Compared with the 2.0-mm general mesh finest model, the total strain energy values were − 0.01% and − 0.33% for the 3.0- and 4.0-general mesh models, respectively. The analysis models had approximately 1,000,000 elements, 150,000 shells, and 200,000 nodes. The material properties of the bones and joints were established based on previous studies (Table 1) [20–22]. Material properties were assigned using phantomless calibration based on CT Hounsfield units obtained from the clinical CT images. The CT scans were acquired using the same scanner and imaging protocol to maintain consistency in density calibration. The hip joint space was filled with joint-filling elements, and the contact interfaces were defined between these elements and the acetabulum. The friction coefficient on the contact surfaces was set to 0.0, which allowed free movement in the direction parallel to the surface. The sacroiliac joint was also filled with joint-filling elements and modeled as a fixed interface between the sacrum and ilium. The sacrum was fully constrained at the center to approximate the continuity of the pelvic ring in a full-pelvis configuration. The anterior side of the pelvic ring was constrained through a cube, which mimicked the pubic symphysis (Fig. 2a). The pubic symphysis was constrained only in the direction perpendicular to the transection plane.

**Table 1:** Material properties

	Young's modules E (MPa)	Poisson's ratio μ	Yield stress σ_y_ (MPa)	Critical stress σ_c_ (MPa)	Model representation
Bone	1530.6ρ_HA__*_^1.9213^	0.3	116.64ρ_HA_^1.8952^	0.8σ_y_	
Femur (In Analysis 1,2)	1530.6ρ_HA__*_^1.9213^	0.3	10^20**	10^20**	
Sacrum (In Analysis 2)	1530.6ρ_HA__*_^1.9213^	0.3	10^20**	10^20**	
Hip joint	10.3	0.4	−	−	Joint-filling elements and contact interface
Pubic symphysis	397	0.3	−	−	Constraint cube
Sacroiliac joint	150	0.2	−	−	Joint-filling elements and fixed interface

^*^ρHA: HA-equivalent density (g/cm^3^)

^**^10^20: Yield stress and critical stress were set to 10^20^ Mpa to prevent failure in these regions

### Analysis 1: simulation of injury mechanisms

**Fig. 3:**
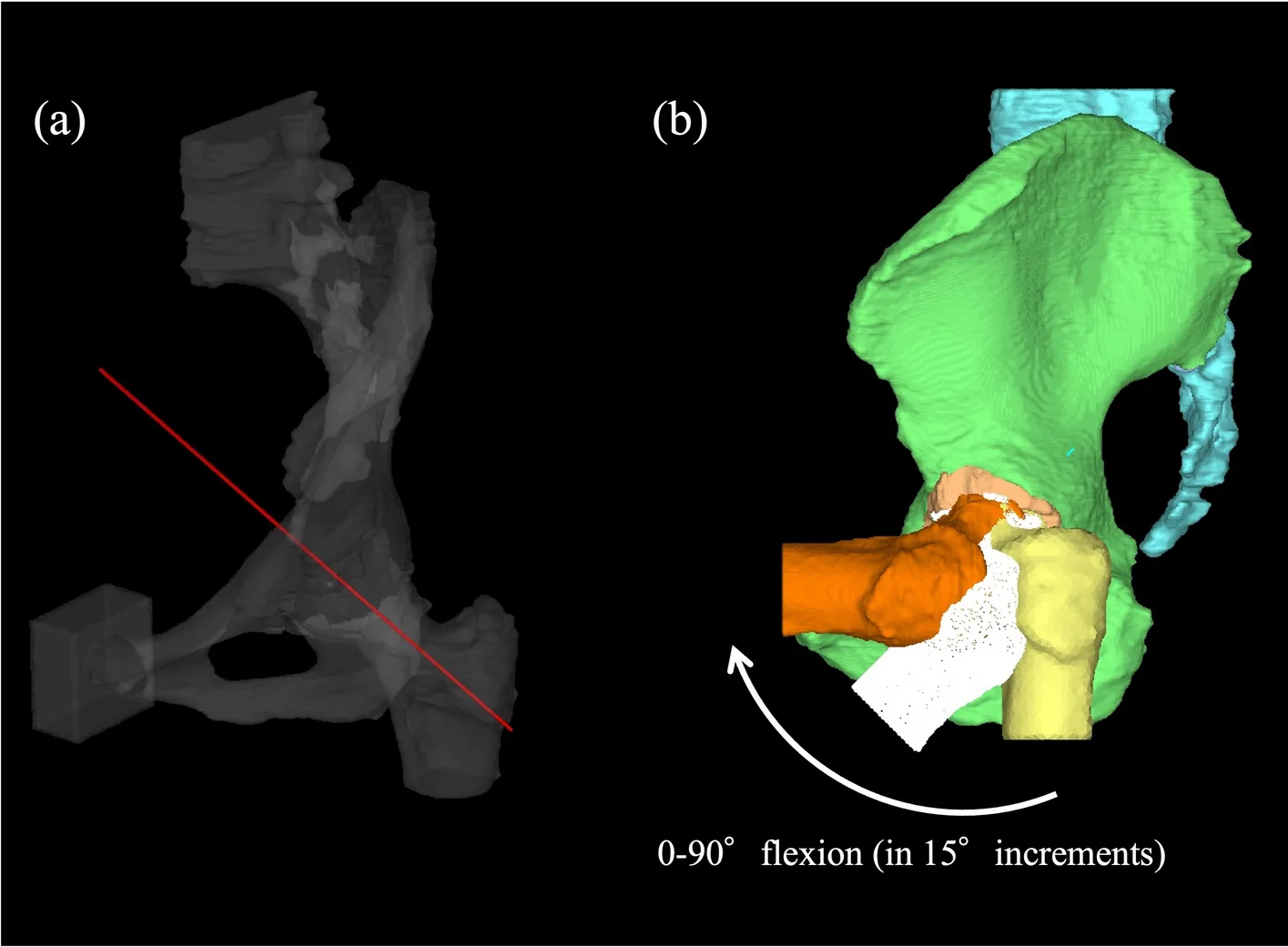
Loading condition of indirect lateral load from the greater trochanter along the axis of the femoral neck in analysis 1. **a** Anterosuperior oblique view demonstrating the loading direction along the axis of the femoral neck. **b** The hip flexion positions from 0º (white) to 90º (orange) at 15º increments

To assess fracture patterns based on the loading site, load conditions were established according to the injury mechanisms described in the Young and Burgess classification. Based on the Young and Burgess classification, we decided to consider external forces in three-dimensional directions: namely horizontal, vertical, and anteroposterior directions. The anterior–posterior compression type was not evaluated, as anterior–posterior compression forces do not occur after falling posterior-laterally from the standing position. The loading conditions of the vertical shear type were established as follows: (1) vertical load originating from the ischial tuberosity simulating a backward fall onto the buttock (Fig. 2b). The loading conditions of the lateral compression [LC] type were established using the following methods (Fig. 2b): (2) direct horizontal load from the iliac wing, (3) indirect horizontal load from the greater trochanter via the hip joint, and (4) indirect lateral load from the greater trochanter along the axis of the femoral neck to the hip joint. In the fourth method, models were created with consideration of hip position (0º, 15º, 30º, 45º, 60º, 75º, and 90º flexion with neutral rotation) (Fig. 3). In this method, the femur was assumed to be high-yield-stress material (yield stress = 10^20^ MPa) to prevent the influence of femoral osteoporosis, which could lead to femoral neck or trochanter fractures. The femur was completely constrained, except the loading direction.

In each analysis, loads were applied incrementally up to 2000 N in 40 N increments. The Newton–Raphson method was used to perform material nonlinear analysis. If the maximum principal stress reaches the critical stress, each element starts tensile failure. The critical stress of the bone was defined as 0.8 times the yield stress [23]. According to previous studies [23–26], if the Drucker–Prager equivalent stress [27] reaches the yield stress, each element starts plastic deformation. If the absolute value of the elements’ minimum principal strain exceeds the crushing strain, the element could fail in compression. Element failure itself was not regarded as equivalent to clinical fracture. Instead, a fracture-like failure pattern was defined as the formation of a contiguous cluster of compressive and tensile failure elements along an anatomically plausible fracture path. The load and failure elements’ distribution were evaluated at the first analysis step in which such a contiguous failure-element cluster was observed. The distribution of compressive and tensile failure elements in each model was compared.

### Analysis 2: failure load analysis of the ilium based on hip position

**Fig. 4:**
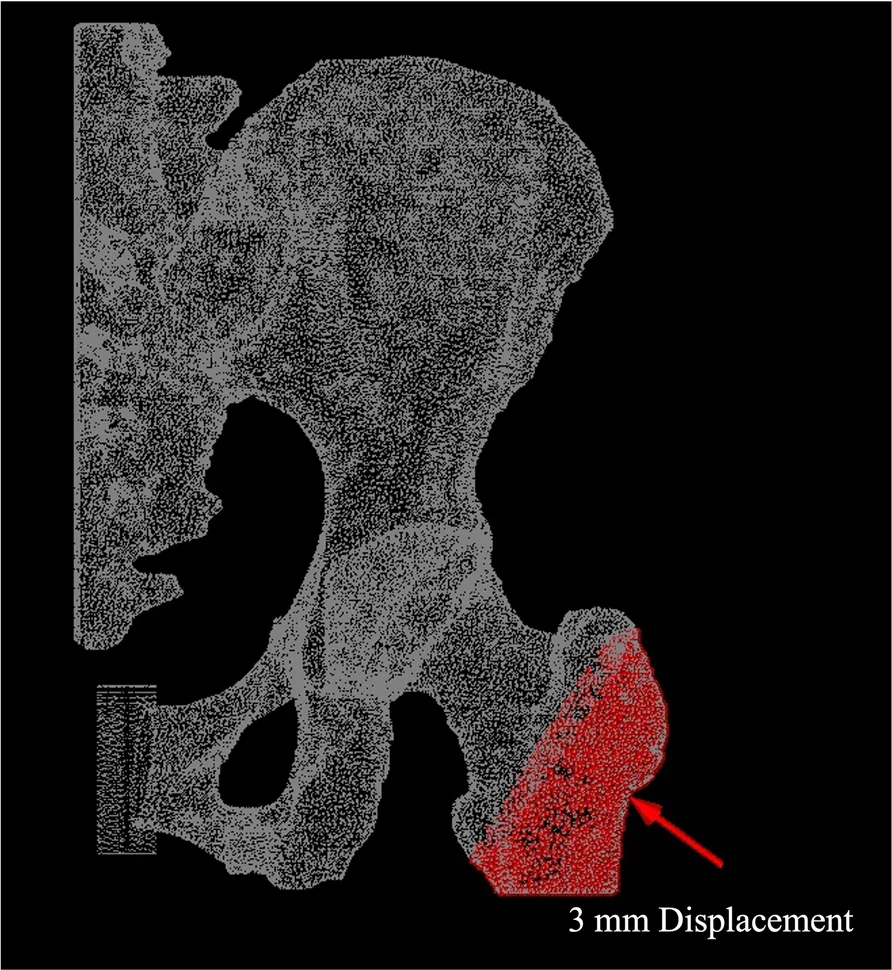
Displacement conditions in analysis 2. A 3.0-mm displacement was applied through the femur

Based on the results of analysis 1, the method of applying indirect lateral load from the greater trochanter along the axis of the femoral neck with consideration of hip flexion was selected. Then, a strength assessment was performed to evaluate the failure load of the ilium in various hip positions in detail. In particular, a 3.0-mm displacement was applied to the models corresponding to each hip position (0º, 15º, 30º, 45º, 60º, 75º, and 90º flexion) that was associated with neutral rotation, 25º internal rotation, and 25º external rotation. In total, 21 combinations comprised seven hip flexion positions, and three rotation positions were analyzed. These models were categorized into three groups according to the rotational hip position: the 0º rotation group, the 25º external rotation group, and the 25º internal rotation group. The femur and sacrum were assumed to be high-yield-stress materials (yield stress = 10^20^ MPa). The femur was displaced 0.15-mm per step in the direction of the femoral neck axis (Fig. 4). Using the vertical axis representing the external constraint force and the horizontal axis representing the displacement steps, the force–displacement curves were created. The peak value of the force–displacement curve was used to calculate failure load. In all models in analysis 2, the sacrum and femur were set as high-yield-stress materials. Therefore, the external constraint force corresponded to the total load applied to the ilium, and its peak value corresponded to the load value when element failure occurs in the ilium.

## Results

### Results 1: simulation of injury mechanisms

**Fig. 5:**
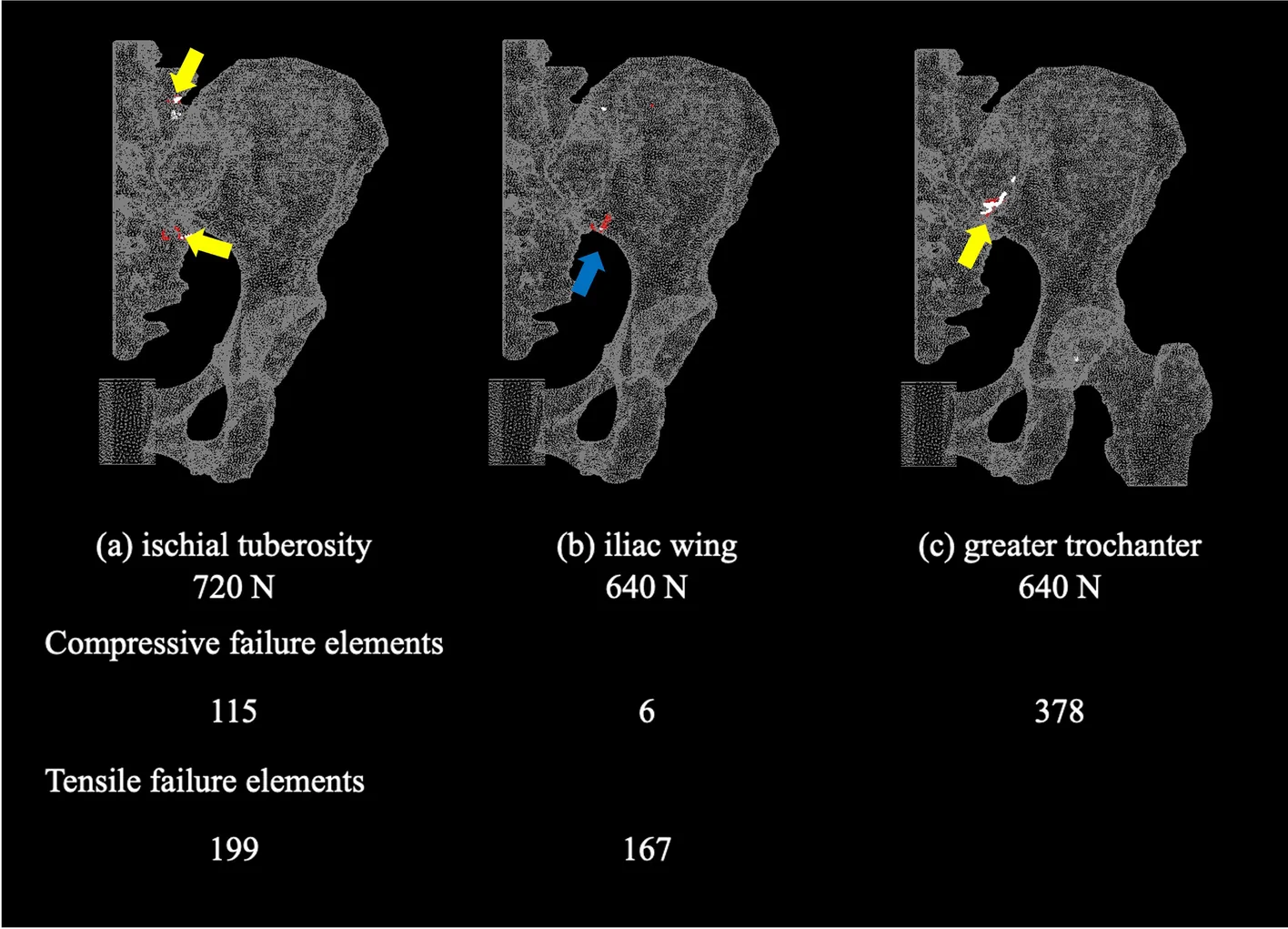
Results of direct loading conditions in Analysis 1. White elements indicate compressive failure elements and red elements indicate tensile failure elements. Blue arrows indicate iliac failure clusters, whereas yellow arrows indicate sacral failure clusters. The numbers below each image indicate the number of compressive and tensile failure elements. **a** Vertical loading from the ischial tuberosity. **b** Horizontal loading from the iliac wing. **c** Horizontal loading from the greater trochanter

**Fig. 6:**
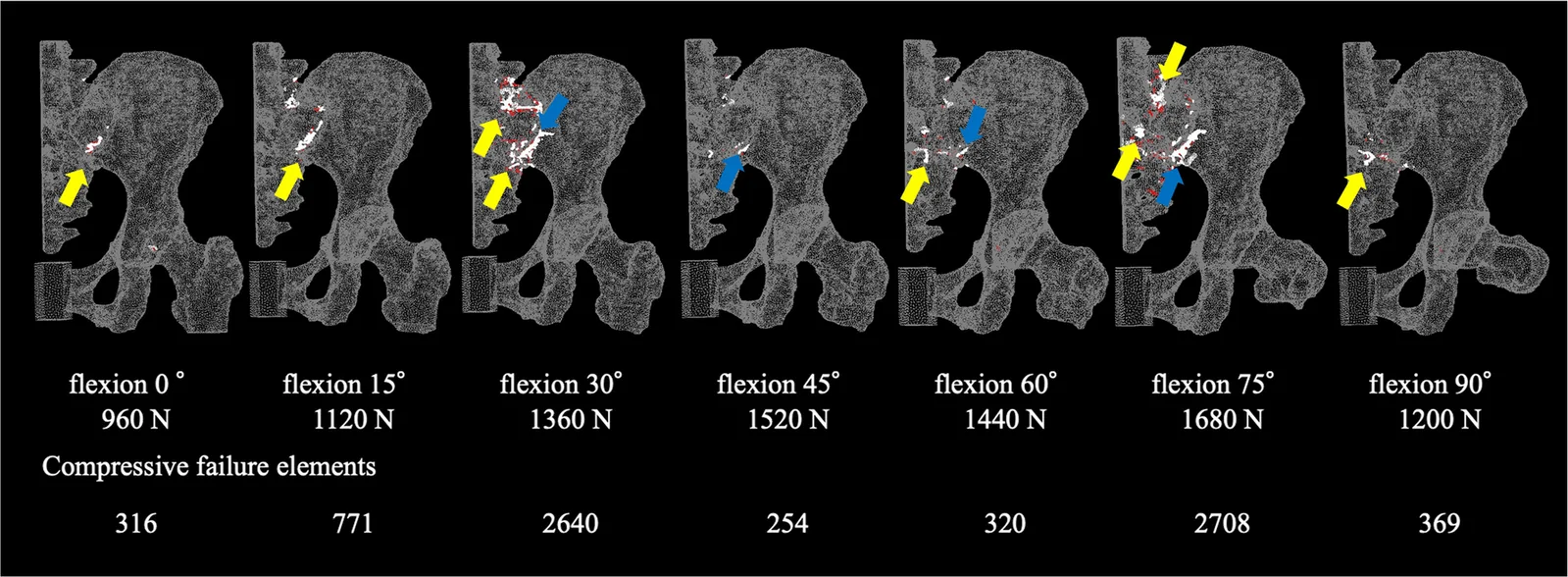
Results of indirect lateral loading conditions from the greater trochanter along the axis of the femoral neck in Analysis 1. Color coding and arrows are the same as in Fig. 5. The numbers below each image indicate the number of compressive failure elements

**Fig. 7:**
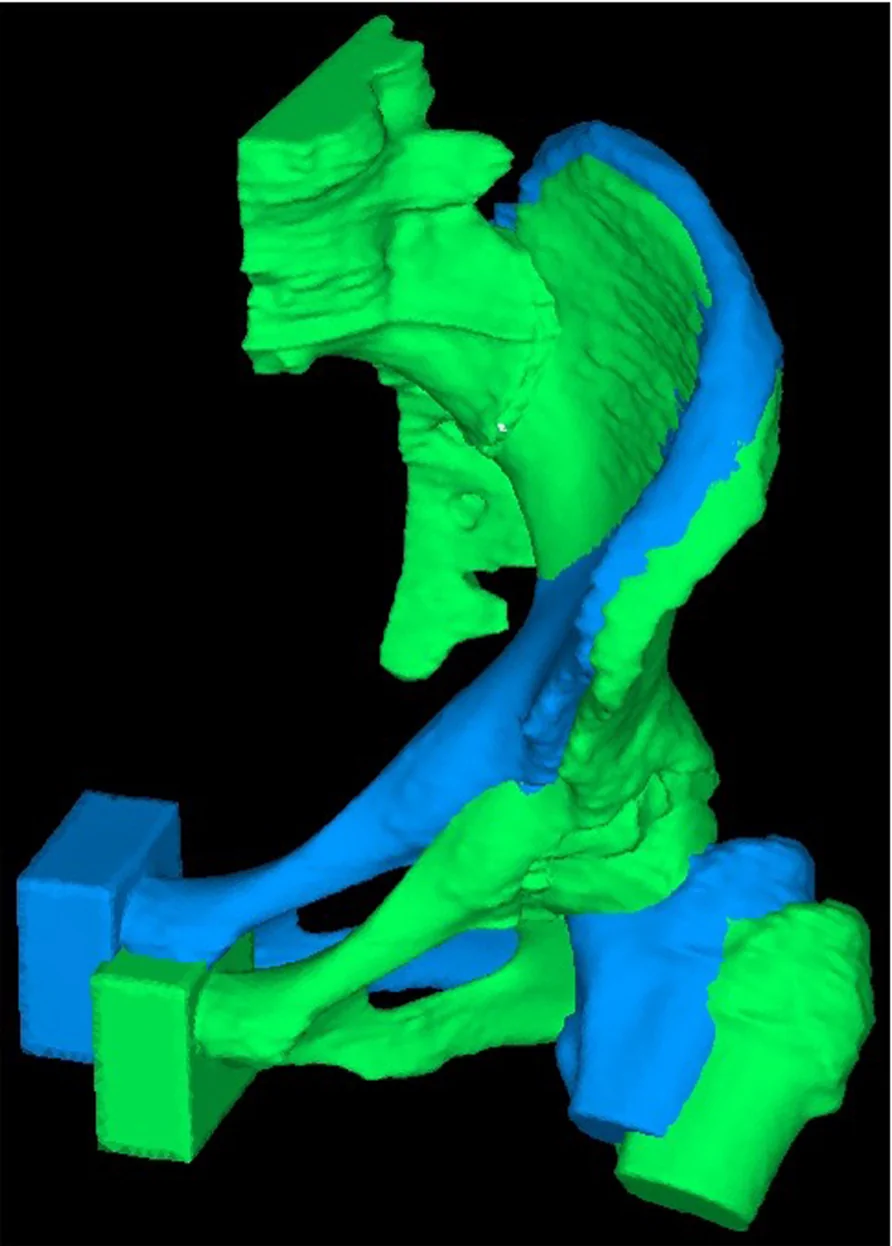
Combined anterosuperior oblique views of the 45º flexion model before (blue) and after (green) loading

Figures 5 and 6 show the fracture-like failure clusters obtained from nonlinear analyses. White elements indicate compressive failure elements, whereas red elements indicate tensile failure elements. In the model of vertical force from the ischial tuberosity (Fig. 5a), horizontal indirect force from the greater trochanter (Fig. 5c), clusters of compressive and tensile failure elements were mainly observed in the sacrum. In contrast, direct horizontal loading from the iliac wing produced clusters of tensile failure elements in the ilium (Fig. 5b). In the models of indirect lateral force from the greater trochanter along the axis of the femoral neck with consideration of hip flexion (Fig. 6), clusters of compressive failure elements were predominantly observed in the iliac wing, particularly at 30º–60º flexion. Figure 7 presents the anterosuperior oblique view of the 45º flexion model, demonstrating external rotational deformation of the iliac wing.

### Results 2: failure load analysis of the ilium according to hip position

**Fig. 8:**
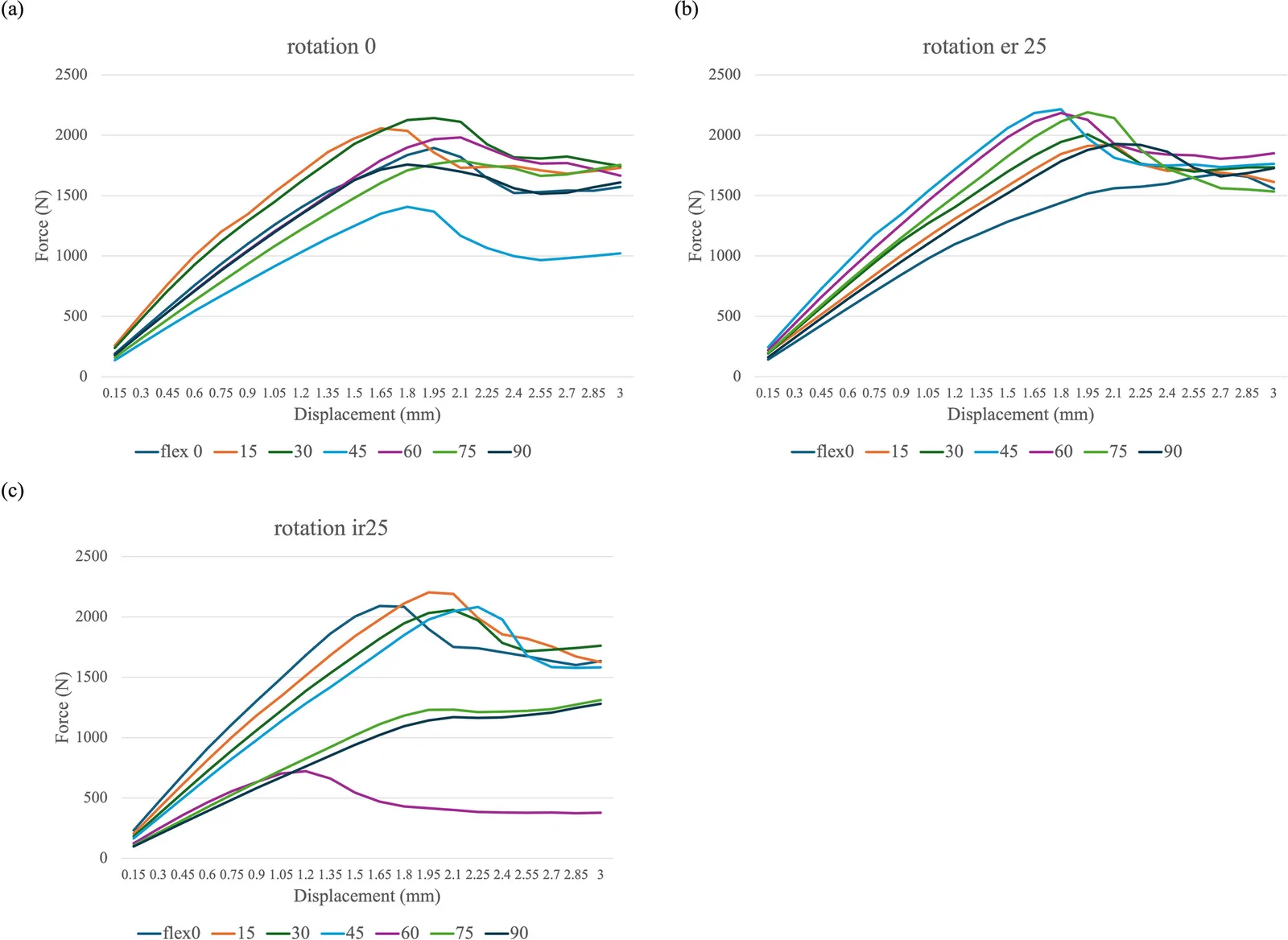
Results of analysis 2. Strength analsis according to hip flexion position. The 0º, 15º, 30º, 45º, 60º, 75º, and 90º models were analyzed according to each rotation group. The force–displacement curves of the (**a**) 0º rotation group, **b** 25º external rotation (er) group, and **c** 25º internal rotation (ir) group are presented

Figure 8 depicts the failure loads of analysis 2. Force–displacement curves were presented for each of the following groups: the 0º rotation group (Fig. 8a), the 25º external rotation group (Fig. 8b), and the 25º internal rotation group (Fig. 8c). Among the models, the 45º flexion model in the 0º rotation group, the 0º flexion model in the 25º external rotation group, and the 60º flexion model in the 25º internal rotation group had the lowest failure load, with forces of 1409, 1682, and 722 N, respectively.

## Discussion

According to the QCT/FEA model, the fracture-like failure mode varied according to the load transmission pathway during lateral compression loading. In addition, the failure behavior of the iliac bone may vary according to the direction of the applied force. Our model, which specifically considered the bone morphology of patients with FFP, suggests that hip position may influence the fracture-like failure patterns during lateral falls. This suggests that not only fall direction but also hip positioning at the time of impact should be considered in strategies aimed at preventing, managing, and treating FFP in elderly patients.

The fracture-like failure mode varied according to the load transmission pathway during lateral compression loading. Iliac fracture-like failure patterns resembling Rommens IIIa FFP may be associated with both direct lateral impact to the ilium and indirect load transmission through the hip joint. Both lateral compression type 2 (LC2) in the Young and Burgess classification and Rommens type IIIa involve iliac fracture [28]. However, LC2 fractures are characterized by internal rotation of the iliac wing caused by direct lateral compression forces to the ilium [9, 29–31], whereas Rommens type IIIa FFP involves external rotation of the iliac wing [29, 32]. These findings suggest that indirect load transmission through the hip joint under specific hip positions may contribute to fracture-like failure patterns distinct from LC2 fracture [33]. To our knowledge, this study is the first to simulate fracture-like failure patterns resembling Rommens type IIIa FFP under specific hip positions using QCT/FEA models.

The mechanical behavior of the iliac bone may vary according to the direction of the applied force. In all models in analysis 2, element failure occurred in the iliac wing along the sacroiliac joint. Therefore, the peak values of the force–displacement curves were used to assess the load value at the onset of iliac wing fracture-like failure. Each model exhibited different peak values in the force–displacement curve, with the lowest peak observed in the 60º flexion model within the internal rotation group. In this study, changes in hip position altered the orientation of the femoral neck axis, which may have modified the loading vector transmitted through the hip joint to the pelvis. Such changes in loading direction, especially hip flexion combined with internal rotation, may have shifted stress concentration patterns within the pelvic ring, thereby altering the susceptibility of the posterior ilium to fracture-like failure. Previous biomechanical studies have reported that fall patterns and injury sites influence the load vectors acting on the femur and hip joint [34, 35]. In addition, previous QCT/FEA studies conducted under physiological loading conditions have shown that stress distributions within the pelvic ring vary depending on the loading conditions [36]. Although previous studies mainly investigated physiological gait loading rather than lateral fall impact, these findings support the concept that loading conditions influence stress transmission within the pelvic ring.

This study had several limitations. First, this was a single-case study of Rommens type IIIa FFP and should therefore be interpreted as a hypothesis-generating biomechanical analysis. Individual variations in pelvic morphology may be associated with fracture-like failure patterns [37]. Second, to simulate the fracture patterns from the actual CT scan images of Rommens type IIIa FFP, this study utilized a hemi-pelvic model with constraints applied at the center of the pelvis, which may not fully reproduce physiological pelvic motion during lateral falls. In addition, the material-property assignment was based on phantomless calibration using clinical CT data, which may introduce uncertainty in density-based material estimation. A whole-pelvis model generated from a mirror image of the intact side may better reproduce physiological pelvic biomechanics. Third, static analyses were conducted in this study. Static analysis has been reported to be useful for evaluating fracture mechanisms in falls among the elderly [23]. On the other hand, it has also been reported that the strain rates associated with falls can significantly reduce the resistance of bone to crack propagation [38], suggesting that future studies should consider dynamic analysis. Fourth, we focused solely on the hip joint and pelvis, thereby disregarding the influence of muscles and ligaments. Finally, the femur was assigned a high yield stress to focus on fracture-like failure patterns in the pelvis. Therefore, the present model may not fully reproduce the interaction between femoral and pelvic failure observed in actual falls. Despite these limitations, the present findings may provide biomechanical insights into possible injury mechanisms of Rommens type IIIa FFP.

## Conclusions

The injury mechanism of Rommens type IIIa FFP may involve not only direct lateral impact to the ilium but also indirect load transmission through the hip joint. In addition, hip position may influence load transmission through the hip joint during lateral falls.

## Data Availability

The data supporting the findings of this study include quantitative computed tomography images, finite element model parameters, and simulation results. Due to the large file sizes and proprietary software formats of finite element models, the complete datasets are not publicly available. However, the data and model parameters necessary to reproduce the analyses are available from the corresponding author upon reasonable request.
